# Unscented Kalman Filter for Brain-Machine Interfaces

**DOI:** 10.1371/journal.pone.0006243

**Published:** 2009-07-15

**Authors:** Zheng Li, Joseph E. O'Doherty, Timothy L. Hanson, Mikhail A. Lebedev, Craig S. Henriquez, Miguel A. L. Nicolelis

**Affiliations:** 1 Department of Computer Science, Duke University, Durham, North Carolina, United States of America; 2 Department of Biomedical Engineering, Duke University, Durham, North Carolina, United States of America; 3 Department of Neurobiology, Duke University, Durham, North Carolina, United States of America; 4 Department of Psychology and Neuroscience, Duke University, Durham, North Carolina, United States of America; 5 Duke Center for Neuroengineering, Duke University, Durham, North Carolina, United States of America; 6 Edmond and Lily Safra International Institute of Neuroscience of Natal, Natal, Brazil; New York University, United States of America

## Abstract

Brain machine interfaces (BMIs) are devices that convert neural signals into commands to directly control artificial actuators, such as limb prostheses. Previous real-time methods applied to decoding behavioral commands from the activity of populations of neurons have generally relied upon linear models of neural tuning and were limited in the way they used the abundant statistical information contained in the movement profiles of motor tasks. Here, we propose an n-th order unscented Kalman filter which implements two key features: (1) use of a non-linear (quadratic) model of neural tuning which describes neural activity significantly better than commonly-used linear tuning models, and (2) augmentation of the movement state variables with a history of *n-1* recent states, which improves prediction of the desired command even before incorporating neural activity information and allows the tuning model to capture relationships between neural activity and movement at multiple time offsets simultaneously. This new filter was tested in BMI experiments in which rhesus monkeys used their cortical activity, recorded through chronically implanted multielectrode arrays, to directly control computer cursors. The 10th order unscented Kalman filter outperformed the standard Kalman filter and the Wiener filter in both off-line reconstruction of movement trajectories and real-time, closed-loop BMI operation.

## Introduction

Research on brain-machine interfaces (BMI) – devices that directly link the brain to artificial actuators [1], [2], [3] – has experienced rapid development during the last decade primarily because of the expectation that such devices may eventually cure severe body paralysis caused by injury or neurodegenerative disease [4], [5], [6], [7], [8]. A core component of BMIs is the computational algorithm that decodes neuronal activity into commands that drive artificial actuators to perform movements at the operator's will. Signal processing and machine learning techniques have been applied to the problem of inferring desired limb movements from neural recordings [9]. These include the population vector method [10], [11], [12], [13], [14], [15], [16], the Wiener filter [3], [17], [18], [19], [20], the Kalman filter [21], [22], [23], [24], the particle filter [25], [26], [27], [28], point process methods [29], [30], [31], [32], artificial neural networks [18], [33], [34], [35], and discrete state Bayesian approaches [18], [36], [37], [38]. Decoding methods using linear models of the relationship between neural activity and limb movements, such as the Wiener filter and Kalman filter, are most commonly used in experimental research on BMIs. These methods cannot handle non-linear models, which describe neuronal modulations better but require more complex algorithms such as the particle filter [39], a non-parametric recursive Bayesian estimator. However, along with the power of particle filters comes a heavy computational cost, which makes this approach difficult to implement in real-time BMI systems. The space of possible non-linear models is vast, and selecting an appropriate model – one that offers significant improvement over a linear model while avoiding “over-fitting” of parameters [40] – is a non-trivial task. Combined with the more difficult software engineering involved, these factors explain the rarity of non-linear models in real-time BMI implementations.

We propose a new computational approach for BMIs, the n-th order unscented Kalman filter (UKF), to improve the extraction of motor commands from brain activity. Our experiments showed that this new approach offers more accuracy compared to methods which use linear models while remaining computationally light enough for implemention in real-time. This filter offers three improvements upon previous designs of BMI decoding algorithms. First, our filter allows the use of non-linear models of neuronal modulations to movements (*neural tuning models*). Our experiments demonstrate the increased accuracy of our quadratic model versus the previously-used linear model. Second, our filter takes advantage of the patterns of movements performed during the execution of tasks. For example, a prosthetic used to aid in feeding has to perform a stereotypical pattern of movements: the prosthetic actuator moves back and forth between the user's mouth and the food items placed on a tray. Our approach uses this stereotypic pattern to improve BMI output accuracy. Third, our filter allows the relationships between neural activity and arm movement at multiple time offsets to be used simultaneously.

These improvements were facilitated by extending the Kalman filter in two ways. First, the unscented Kalman filter [41], which uses a non-stochastic simulation method to approximate non-linear function evaluation on random variables, was used to allow non-linear neural tuning models. Second, the state of our filter was extended to keep a history (of length n) of the desired hand movements to allow an autoregressive (AR n) movement model and neural tuning to all n consecutive time offsets. These two elements were combined in a system that is relatively simple, robust, and fast enough for real-time, closed-loop BMI applications.

Our algorithm was tested both off-line and in real-time, closed-loop experiments in which cortical recordings were obtained from macaque monkeys (*Macaca mulatta*) trained to perform two reaching tasks. In off-line comparisons, our method demonstrated significantly better accuracy compared to the Kalman filter, the Wiener filter, and the population vector method [10], [13]. In on-line, closed-loop BMI control, the monkeys followed targets significantly better when using our method than when using the Kalman or the Wiener filter.

## Results

### Behavioral Tasks and Cortical Recordings

**Figure 1:**
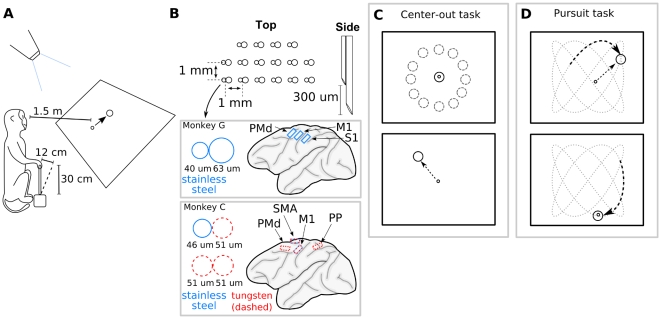
Schematics of the experimental task and cortical implants. A: The cursor and the visual targets were projected to the screen mounted 1.5 m in front of the monkey, and the monkeys moved the cursor with a hand held joystick with length 30 cm and maximum deflection 12 cm. The monkeys received fruit juice rewards when they placed the cursor inside targets. B: Microwire electrode array diagram (top) and schematics of the placement of the arrays in the cortex of two monkeys. C: Schematics of the center-out task. After holding the cursor at the screen center, the monkeys moved it to a peripheral target that appeared at a random angle and a fixed radius from the center D: Schematics of the pursuit task. The monkeys tracked a continuously moving target whose trajectory was a Lissajous curve.

We trained 2 rhesus macaques (Monkey C and Monkey G) to perform reaching tasks that incorporated stereotypic patterns of movements. The monkeys manipulated a hand-held joystick to acquire visual targets with a computer cursor (Figure 1A). In the *center-out task*, the cursor was moved from the screen center to targets randomly placed at a fixed radius around the center (Figure 1C). In the *pursuit task* the monkeys tracked a continuously moving target which followed a Lissajous curve (Figure 1D).

Both monkeys were implanted with multielectrode arrays in multiple cortical areas. Monkey C was implanted in M1, PMd, posterior parietal cortex (PP) and supplementary motor area (SMA) in the right hemisphere. Monkey G was implanted bilaterally in primary motor cortex (M1), primary somatosensory cortex (S1) and dorsal premotor cortex (PMd). Extracellular discharges of 94 to 240 (average 142) cortical neurons were recorded while each monkey performed the behavioral tasks.

We applied the n-th order unscented Kalman filter to the data collected in 16 daily sessions: 6 sessions from Monkey C and 10 sessions from Monkey G. Data used from each session ranged from 9 to 25 minutes. After evaluating filter accuracy off-line, we conducted six on-line experiments, three with each monkey, while the monkeys controlled the BMI using the unscented Kalman filter and comparison methods in closed-loop operation. We treated the neurons recorded from different cortical areas as one ensemble; differences between individual cortical areas were not considered here.

### N-th Order Unscented Kalman Filter

**Figure 2:**
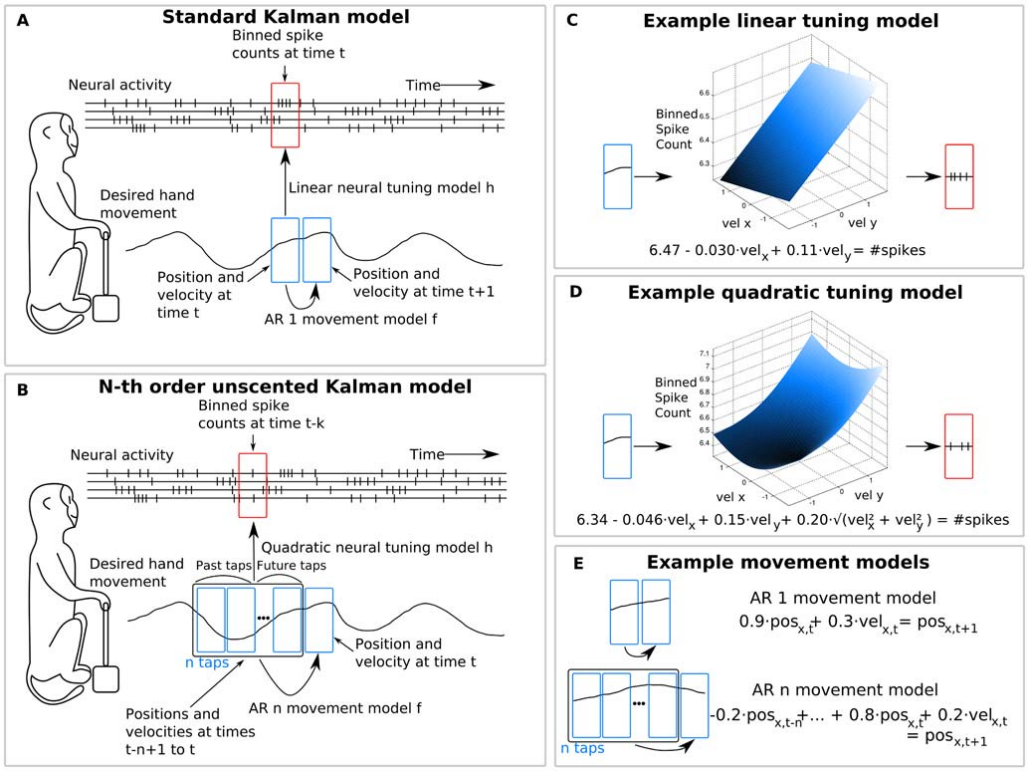
Comparison of the standard Kalman filter with the n-th order unscented Kalman filter. A: The standard Kalman filter predicts future position and velocity based on a linear model of neural tuning and predictions of the present position and velocity only. B: The n-th order unscented Kalman filter predicts future position and velocity based on a quadratic model of neural tuning and n history taps of position and velocity (AR n). C: Example of linear neural tuning model. D: Example of quadratic tuning model. E: Example AR 1 and AR n movement models.

Our n-th order unscented Kalman filter (UKF) combined two extensions to the standard Kalman filter [42]: (1) the unscented transform [41], which allowed approximate filtering under non-linear models, and (2) the n-th order extension, which allowed autoregressive movement models and multiple temporal-offset neural tuning models. Figure 2 shows a comparison of the standard Kalman filter (Figure 2A) and the n-th order unscented Kalman filter (Figure 2B), as well as examples of a linear neural tuning model (Figure 2C), quadratic neural tuning model (Figure 2D), and autoregressive (AR 1 vs AR n) movement models (Figure 2D). A side-by-side comparison of the filtering equations is shown in Table 1.

**Table 1:** Comparison of the equations for the standard Kalman filter and our unscented Kalman filter.

	Kalman filter	Unscented Kalman filter
*Predict step*	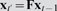	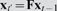
	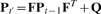	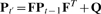
*Update step*		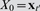
		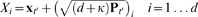
		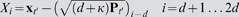
		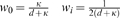 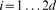
		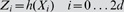
	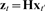	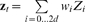
		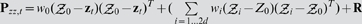
	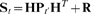	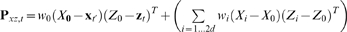
	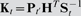	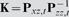
	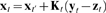	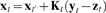
	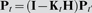	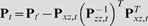

Like the standard Kalman filter, the n-th order unscented Kalman filter inferred the hidden state (the position and velocity of the desired movement) from the observations (neuronal rates). The state transition model or *movement model*, predicted the hidden state at the current time step given the state at the previous *n* time steps. The observation model or *neural tuning model* predicted the expected neuronal rates from the estimated desired movement via a non-linear function. We incorporated multiple taps of the state in the neural tuning model to relate neural activity with hand kinematics at multiple time offsets simultaneously. We used a nonlinear *quadratic model of tuning* to express neuronal rates as a function of hand position and velocity.

### Tuning Model Validation

We analyzed the predictive accuracy of the quadratic tuning model used in our n-th order unscented Kalman filter. Firing rates of single neurons were predicted from hand position and velocity using the quadratic (with n = 1 and n = 10 taps) and the linear neural tuning models after the models were fit with linear regression using the Moore-Penrose pseudoinverse. A 10-fold cross-validation procedure was used to test predictive accuracy from 16 recording sessions with an average of 142 neurons recorded per session, and we report results using signal-to-noise ratios (SNR, where the signal was the recorded binned spike count) and correlation coefficients (CC). The n = 1 tap quadratic model (SNR = 0.03±0.29 dB, CC = 0.10±0.09; mean±standard deviation) was more predictive (P<0.001, two-sided, paired sign-test) than the linear model (SNR = 0.01±0.27 dB, CC = 0.07±0.08). 1753 out of 2273 units (approximately 77%) were better predicted using the quadratic model. The n = 10 tap quadratic model (SNR = 0.05±0.32 dB, CC = 0.11±0.10) was more predictive (P<0.001) than the n = 1 tap quadratic model (about 900 or approximately 40% of units were better predicted).

**Figure 3:**
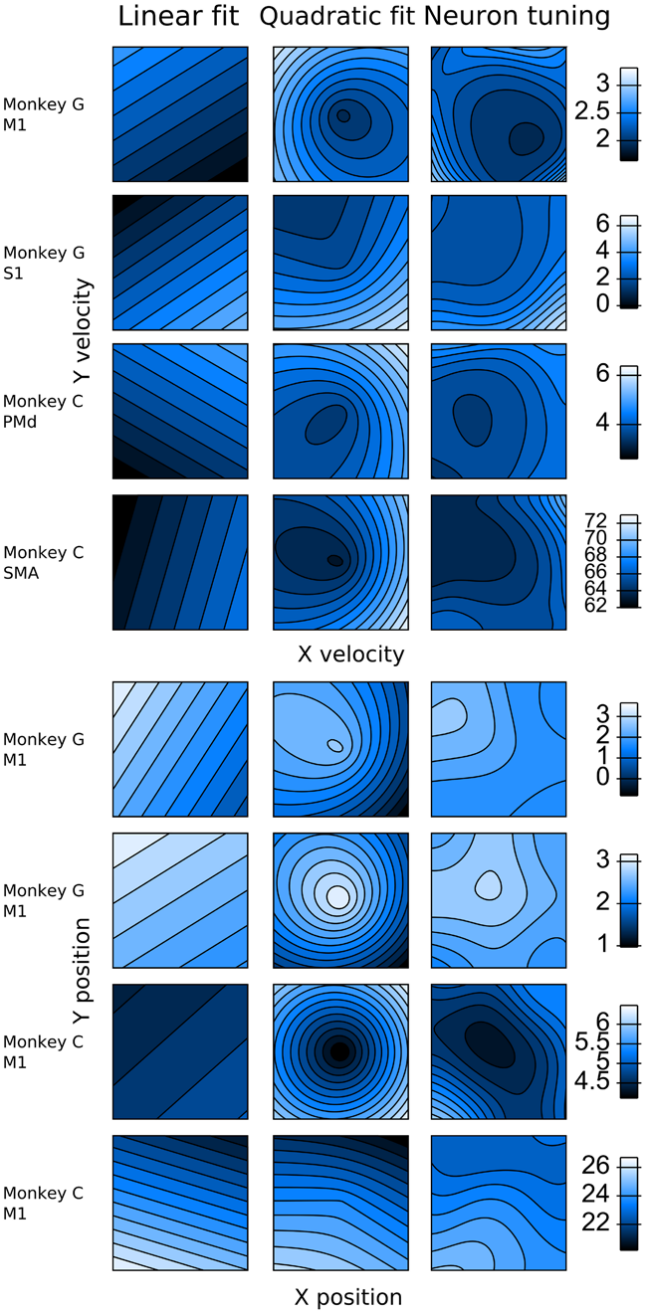
Contour plots of parameter fits for linear and quadratic tuning models to the tuning of eight representative neurons. The plot axes are the x- and y-axis of the hand position or velocity. Brighter intensity of shading indicates higher firing rate, in spikes/sec. The right-most column depicts the smoothed true firing rate. The quadratic model captures the trends of neuronal modulations better than the linear model for most neurons.

The superior performance of the quadratic tuning model is illustrated in the contour plots of Figure 3, which show the tuning to position and velocity of eight representative neurons and parameter fits using the linear and quadratic (n = 1) models. The x and y coordinates in the plots indicate x and y positions or velocities and the brightness of the shading indicates the predicted firing rate (Figure 3, left two columns) and true firing rate (Figure 3, right-most column). For clarity, the fits to velocity (Figure 3, top four rows) and position (Figure 3, bottom four rows) are shown separately. The right-most column of Figure 3 shows the actual firing rate estimated on a 50 by 50 grid, which spanned plus and minus three standard deviations of the position or velocity values (smaller of the standard deviations for x and y) observed during the experimental session, using Gaussian kernel smoothing, with kernel width one standard deviation of the observed values (smaller of the standard deviations for x and y).

For velocity tuning (Figure 3, top four rows), the quadratic model captures the low-center, high-surround tuning pattern seen in many neurons, while the linear model cannot capture this pattern because it is restricted to fitting a plane in the (rate, x, y) space. For position tuning (Figure 3, bottom four rows), the more expressive quadratic model captures the tuning patterns better than the linear model. While more sophisticated models of tuning, such as higher-order or non-parametric models, may model neural activity more accurately, our model is relatively simple, fast to fit and evaluate, and grounded in previous work (see Materials and Methods), while demonstrating significantly better predictive accuracy than the commonly-used linear model.

### Off-line reconstruction

**Figure 4:**
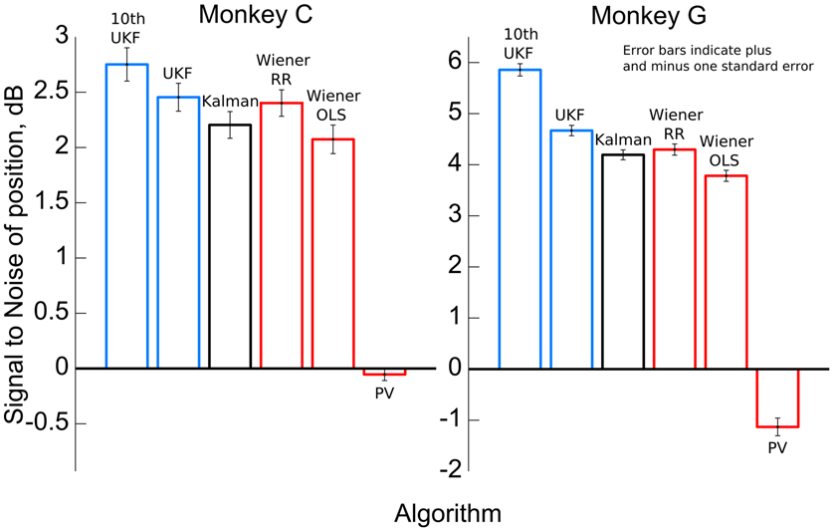
Off-line reconstruction accuracy for 2 monkeys (C and G) for each algorithm. Accuracy is quantified as signal-to-noise ratio (SNR) of the position reconstructions, averaged between x and y dimensions. Error bars indicate plus and minus one standard error.

We compared the ability of our method to reconstruct hand movements from neural recordings with several commonly used, real-time methods by performing 10-fold cross-validation on 16 previously recorded sessions. Parameters for the algorithms were fitted by ridge regression, a regularized form of linear regression, using recorded neural and behavioral (joystick position and velocity) data. The first cross-validation fold of each session was used to optimize ridge regression parameters and omitted from the results. The mean off-line reconstruction accuracy of the 10th order unscented Kalman filter (UKF), the 1st order unscented Kalman filter, the standard Kalman filter, the 10 tap Wiener filter fitted with ridge regression (RR), the 10 tap Wiener filter fitted with ordinary least squares (OLS), and the population vector method used by Taylor et al. are shown in Figure 4, grouped by monkey [13]. The y-axis shows the signal-to-noise ratio (SNR, where the signal was the recorded behavior) of the hand position reconstruction and error bars indicate plus and minus one standard error over the 9 cross-validation folds of each session and the x and y axes (for a total of 108 observations for Monkey C and 180 observations for Monkey G). Reconstruction accuracy for position and velocity, measured in SNR and correlation coefficient, for the algorithms are shown in Table 2, grouped by behavioral task.

**Table 2:** Off-line reconstruction accuracy for the 10th order UKF, Kalman filter, Wiener filter, and population vector method.

Filter	Sessions 1–8 Center-out	Sessions 9–16 Pursuit	Mean difference from KF
Position: SNR • CC			
10^th^ UKF	**3.24±0.16** • **0.75±0.01**	**5.84±0.14** • **0.87±0.00**	**1.51** • **0.05**
1^st^ UKF	2.82±0.14 • 0.73±0.01	5.05±0.13 • 0.85±0.01	0.90 • 0.02
KF	2.23±0.14 • 0.71±0.01	3.83±0.14 • 0.83±0.01	0.00 • 0.00
WF RR	2.58±0.11 • 0.68±0.01	4.19±0.12 • 0.78±0.01	0.35 • −0.04
WF OLS	2.29±0.11 • 0.67±0.01	3.97±0.11 • 0.77±0.01	0.10 • −0.05
PV	−1.31±0.13 • 0.34±0.01	−2.24±0.23 • 0.42±0.01	−4.81 • −0.39
Velocity: SNR • CC			
10^th^ UKF	**1.15±0.06** • **0.50±0.01**	**1.46±0.05** • **0.55±0.01**	**0.11** • **0.03**
1^st^ UKF	**1.15±0.06** • 0.48±0.01	1.42±0.05 • 0.52±0.01	0.09 • 0.01
KF	1.05±0.06 • 0.47±0.01	1.34±0.05 • 0.52±0.01	0.00 • 0.00
WF RR	0.68±0.06 • 0.44±0.01	1.10±0.04 • 0.49±0.01	−0.31•−0.03
WF OLS	0.42±0.06 • 0.42±0.01	0.84±0.04 • 0.47±0.01	−0.56•−0.05
PV	−0.51±0.08 • 0.36±0.01	−0.74±0.13 • 0.41±0.01	−1.82•−0.11

Each cell shows the SNR and CC mean±standard error of 144 data points. The last column shows the mean difference of each algorithm compared against the Kalman filter, where larger numbers are better. WF RR is the 10 tap Wiener filter fitted with ridge regression. WF OLS is the 10 tap Wiener filter fitted with ordinary least squares. Bold numbers indicate the best value in each column.

In terms of position estimates, the 10th order UKF with our quadratic tuning model was consistently more accurate than the other algorithms. The two-sided, paired sign test with 288 observations (16 sessions, 9 folds, 2 dimensions) and significance level 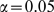 was used to evaluate significance. The 10th order UKF produced position estimates with significantly higher SNR than the 1st order UKF (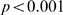, mean difference 0.85 dB), the standard Kalman filter (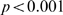, mean difference 1.25 dB), the 10 tap Wiener filter fit using ridge regression (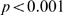, mean difference 1.11 dB), the 10 tap Wiener filter fit using ordinary least squares (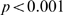 mean difference 1.55 dB), and Taylor's variant of the population vector method (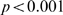, mean difference 5.42 dB). When sessions of pursuit task and center-out task were separately analyzed, the 10th order UKF was 1.23 dB more accurate than the 1st order UKF in the pursuit task and 0.48 dB more accurate in the center-out task.

The 1st order UKF produced position estimates with significantly higher SNR than the standard Kalman filter (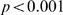, mean difference 0.39 dB), the 10 tap Wiener filter fit using ridge regression (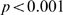, mean difference 0.25 dB), the 10 tap Wiener filter fit using ordinary least squares (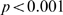, mean difference 0.70 dB), and Taylor's variant of the population vector method (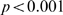, mean difference 4.57 dB).

For predicting velocity, the 10th order UKF produced estimates with significantly higher SNR than the 1st order UKF (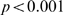, mean difference 0.27 dB), the standard Kalman filter (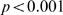, mean difference 0.36 dB), 10 tap Wiener filter fit using ridge regression (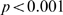, mean difference 0.29 dB), the 10 tap Wiener filter fit using ordinary least squares (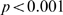 mean difference 0.82 dB), and Taylor's variant of the population vector method (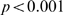, mean difference 2.60 dB).

The 1st order UKF produced velocity estimates with significantly higher SNR than the standard Kalman filter (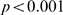, mean difference 0.09 dB), the 10 tap Wiener filter fit using ordinary least squares (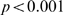 mean difference 0.55 dB), and Taylor's variant of the population vector method (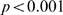, mean difference 2.33 dB).

Similar results were obtained when the correlation coefficient was used as a measure of filter performance.

### On-line performance

We compared the 10th order UKF to the Kalman filter and Wiener filter in on-line, closed-loop BMI control in six recording sessions: three with monkey C and three with monkey G. In each session, the monkey first performed the pursuit task using joystick control for 6 to 10 minutes. During this time period, 5 minutes of data was used to fit parameters for the algorithms. In each session, all algorithms were fit on the same data. Then the monkey performed the pursuit task using BMI control with each algorithm in turn for 5 to 8 minutes. The evaluation order of the algorithms was switched between sessions, however not all orderings could be used in the three sessions for each monkey. During BMI control, the monkey was required to hold the joystick as an indication of active participation; time periods when the monkey did not hold the joystick were omitted from the analysis.

**Figure 5:**
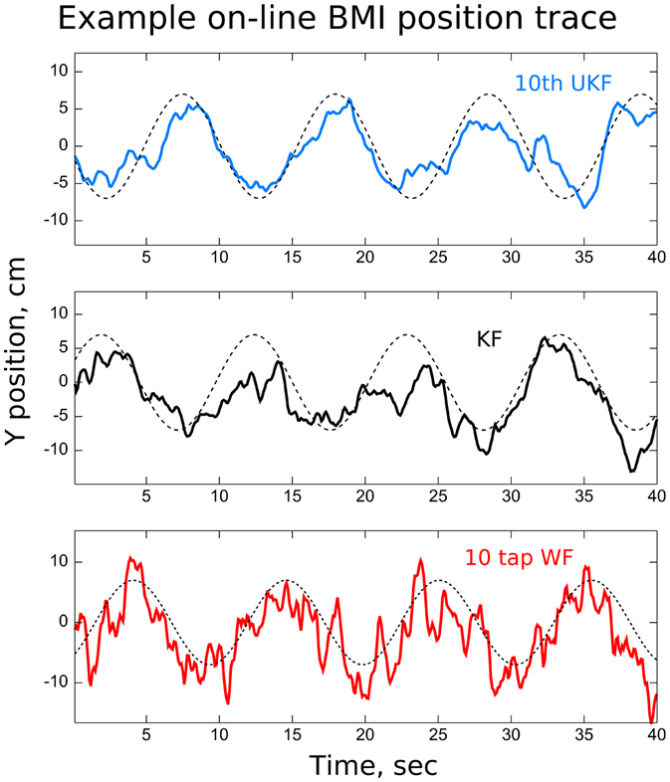
Example traces of y-position during on-line, closed-loop BMI operation in a representative experimental session (session 19, Monkey C). The dashed sinusoidal curves indicate target position.

Performance was measured by comparing the position of the target (the signal for SNR calculations) and the BMI-controlled cursor. Table 3 shows the signal-to-noise ratio and correlation coefficient for each algorithm in each session, with the mean taken across the x and y-axis. Figure 5 shows example traces of the BMI-controlled cursor and target positions in session 19. The two-sided, paired sign-test was used to measure significance with the two axis treated separately and significance value was set at 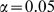. In terms of SNR, the monkeys performed significantly better when using the 10th order UKF than when using the Kalman filter (*p*<0.05, 12 observations) and 10 tap Wiener filter fitted with ridge regression (*p*<0.05, 10 observations). In terms of CC, no comparison was significantly different at the 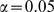 level.

**Table 3:** Comparison of behavioral performance using on-line, closed-loop BMI driven by a 10th order UKF, a Kalman filter, and a 10 tap Wiener filter fit using ridge regression.

Session	Monkey	10^th^ UKF	KF	WF RR
SNR, dB • CC				
17	C	**2.70** • **0.69**	0.70 • 0.47	NA
18	C	**2.73** • **0.72**	2.42 • 0.60	−1.13 • 0.54
19	C	**2.51** • **0.71**	0.80 • 0.53	0.07 • 0.68
20	G	−2.12 • 0.10	**−1.49** • **0.15**	−3.23 • 0.07
21	G	**1.58** • 0.56	1.55 • 0.57	0.77 • **0.58**
22	G	**3.23** • **0.71**	0.39 • 0.48	−0.06 • 0.47
Mean difference from KF		**1.04** • **0.12**	0.00 • 0.00	−1.45 • 0.00

Performance was measured as signal-to-noise ratio and correlation coefficient of the BMI-controlled cursor position to the target position. The bottom row shows mean difference of each algorithm compared against the Kalman filter, where larger numbers are better. Bold numbers indicate the best value in each row.

### Model, parameter, and algorithm analysis

**Figure 6:**
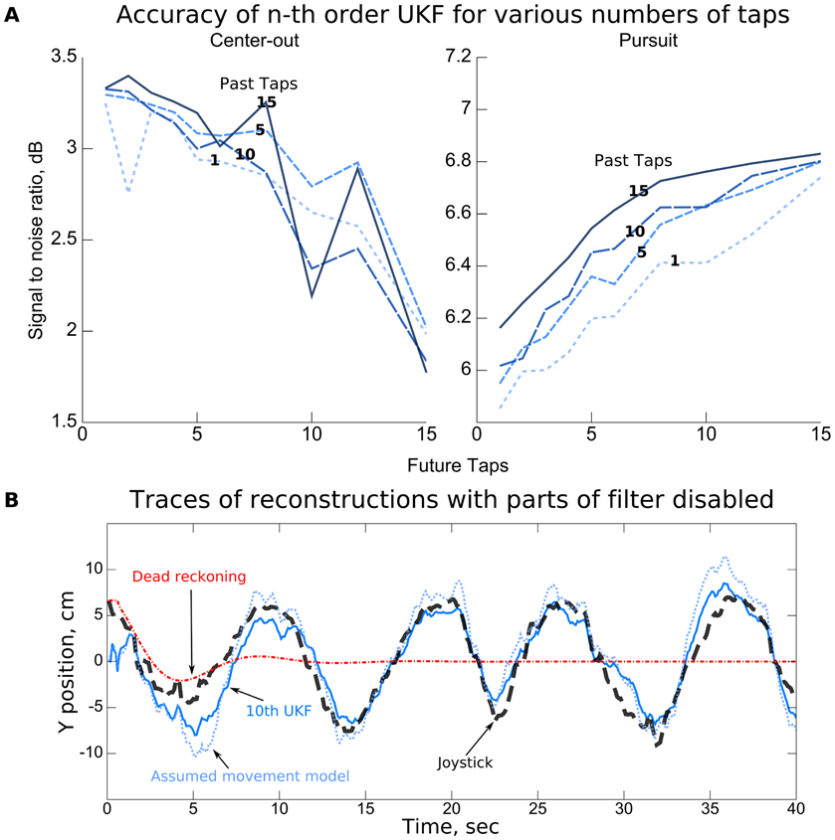
Dependency of reconstruction accuracy on the filter parameters. A: Reconstruction accuracy quantified as signal-to-noise ratio (SNR) versus number of future (x-axis) and past taps (curves). B: Example traces of position reconstruction with parts of filter disabled. The thick dashed curve shows the joystick x-axis position. The solid curve shows the reconstruction using the fully-functional 10th order UKF. The dotted curve shows the reconstruction using a 10th order UKF with the movement model assumed to be the physical equations relating position and velocity, instead of fitted to data. The dash-dotted curve shows the 10th order UKF with the neural observations ignored.

Our neural tuning model related neural activity with behavior both prior to and after the time instant of neural activity. The parameters *past taps* and *future taps*, in units of 100 ms, described the time offsets prior to and after the instant of neural activity between which tuning was modelled, respectively (see Materials and Methods). We investigated the relationship between choices of the number of future and past taps and reconstruction accuracy for the n-th order UKF (Figure 2B). The ridge regression parameter was optimized for each setting of the number of taps using the first fold of 10 fold cross-validation, and we report the accuracy on the remaining 9 folds. Plots of the mean position accuracy over various choices of the number of future and past taps for two sessions, one with center-out task (session 1) and one with pursuit task (session 16), are shown in Figure 6A. The number of future taps is shown on the x-axis and each setting of past taps is depicted as a separate curve. For the pursuit task, the performance steadily increases with the number of future taps and increases slowly with the number of past taps. For the center-out task, the performance was maximum when 15 future and 2 past taps were used. A large number of future taps resulted in decreased performance, while the number of past taps had small effects on performance.

To test the capacity of the movement model to predict hand trajectories, we conducted two analyses. In the first analysis, the neural tuning model update step of the 10th order UKF was disabled so that the filter ignored neural activity and used only the movement model to “dead reckon.” In the second analysis, the movement model was not fit to the training data but set by assumption so that position was the discrete integral of velocity and velocity remained constant except for noise perturbations. The movement model noise covariance was fit to the data under these assumptions by calculating the mean-squared-error matrix of the residuals when using this movement model to predict next states. Figure 6B shows example traces of reconstruction under these two conditions on pursuit task (session 16). The true position of the joystick is shown by the thick dashed curve. The “dead reckoning” filter (dash-dotted curve) produced useless predictions shortly after filtering began, showing that the movement model could not reconstruct the hand trajectory alone, even though the monkey tried to follow a deterministic Lissajous curve. The 10th order filter with the assumed movement model (dotted curve) produced less accurate predictions than the filter with movement model fitted from the data. The position estimate SNR of the 10th order assumed movement model filter was 3.88±0.27 dB (mean±standard error, 18 observations) for the pursuit task session and 2.89±0.44 dB in the center-out task session. For the fully-functional 10th order UKF, the SNR was 6.25±0.23 dB for the pursuit task session and 4.08±0.36 dB for the center-out task session, showing a large benefit to using a fitted movement model, especially for the pursuit task. The position estimate SNR of the 1st order assumed movement model filter was 1.57±0.76 dB for the pursuit task session and 0.54±0.59 dB for the center-out task session. Since the assumed movement model was not fitted to data and the movement model noise covariances were identical, this difference in performance between the 1st order and 10th order assumed movement model filters must arise from the different accuracies of the 1 tap and 10 tap quadratic neural tuning model. The large difference in accuracy (2.30 and 2.35 dB) shows the benefit of modeling neural tuning across multiple time offsets simultaneously, although much of this benefit likely comes from the autocorrelation of movements, which is also captured by data-fitted movement models.

To quantify the extent the approximations of the unscented Kalman filter affected performance, we performed off-line reconstructions using standard particle filters with identical models as the 1st and 10th order unscented Kalman filter. The particle filters used 50,000 particles and the same parameters, initial conditions, and test data as the unscented Kalman filters. Since we had many sessions and cross-validation folds for comparison, only one particle filter run was performed per session and cross-validation fold. We used the posterior mean of the particles as the output. For the 1st order model, the particle filter produced significantly more accurate position reconstructions (two-sided, paired sign-test, 288 observations, 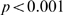, mean difference 0.07 dB) than the unscented Kalman filter. For the 10th order model, the difference in performance was not significant at the 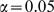
level, with the unscented Kalman filter having a nominal 0.02 dB advantage in mean SNR. This was likely due to the large state space (40 dimensional) associated with the 10th order model—even the large number of particles could not represent distributions in this state space as well as a multivariate normal distribution, hence the UKF provided similar accuracy even with the unscented approximation.

**Figure 7:**
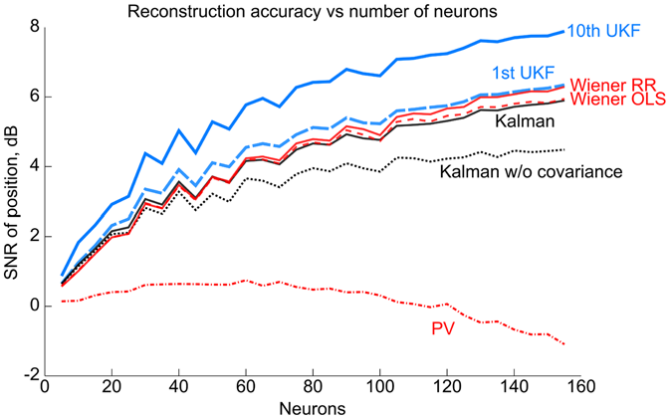
Dependency of reconstruction accuracy for each algorithm on the number of neurons. The y-axis depicts the mean accuracy among 10 random subsets of neurons used by all algorithms to make reconstructions. The curve labeled Kalman w/o covariance indicates the reconstruction accuracy of a Kalman filter with the off-diagonal entries of the neural tuning model noise covariance set to zero.

Figure 7 shows off-line reconstruction accuracy for a pursuit task session when different-sized subsets of the neurons are used (*neuron dropping curves*). For each setting of the number of neurons, 10 subsets of neurons were randomly selected and each algorithm was evaluated on these subsets using 10 fold cross-validation. The first fold was reserved for finding optimal ridge regression parameters, and the mean accuracy on the nine remaining folds are plotted in Figure 7. The 1st and 10th order unscented Kalman filter reconstructs position more accurately than the Kalman filter, Wiener filter, and population vector method even for small numbers of neurons. The advantage of the 10th order UKF increases with the number of neurons. The Wiener filter fitted with ridge regression approaches the accuracy of the 1st order UKF as the number of neurons increases. As expected, the benefit of ridge regression for fitting the Wiener filter grows larger as the number of neurons, and hence number of parameters, increases. Modeling the noise covariance between neurons becomes more important as the number of neurons increases, as can be seen by the lower performance of a modified Kalman filter which does not model neuron noise covariance (Kalman w/o covariance) compared to the unmodified Kalman filter. The neural tuning model noise covariance of the Kalman w/o covariance filter has all entries not on the diagonal set to zero. The population vector method peaks in performance at around 60 neurons and then decreases in accuracy, demonstrating the sub-optimality of the parameter fitting procedure which ignores covariance among neurons.

In terms of computational load, the MATLAB implementation of the 10th order UKF on an Intel Pentium 4 class computer used 0.012±0.005 seconds per iteration (mean±standard deviation), or around 80 Hz on average. The 30th order UKF (15 future and 15 past taps) used 0.0360±0.0001 seconds per iteration, or around 28 Hz on average. Our on-line implementation in C++ using Automatically Tuned Linear Algebra Software (ATLAS) easily executed faster than 10 Hz, our binning frequency.

## Discussion

In this study, we achieved an improvement over previous closed-loop linear BMI decoding by implementing a more accurate decoding algorithm, the n-th order unscented Kalman filter (UKF). This filter modeled arm movement profiles better because it used the history of past movement, and it described neuronal modulations to movements better by using a quadratic model of neuronal tuning which included tuning at multiple time offsets. The filter performed well both in off-line reconstruction of previously recorded data and on-line, closed-loop BMI operation.

### Review of previous algorithms

Much work has been done investigating algorithmic methods for decoding continuous control signals from extracellular neural recordings for neuroprosthetics (for a survey see Bashashati et al. [9]). The underlying theory stems from the pioneering work of Georgopoulos et al. [43], which reported the cosine relationship between firing rates of M1 neurons and the angle between arm movement and the neurons' preferred directions. The observation of this relationship led to a hypothesis of neuronal encoding of movements called the population vector model, in which movement velocity is calculated as vector sums of single-neuron vectors pointing in the neurons' preferred directions and scaled by the neurons' firing rates [10]. Many BMI studies used this approach to decode movement parameters from population activity [11], [12], [13], [14], [15].

The Wiener filter, an optimal linear regression method, improves upon the population vector approach. The Wiener filter has been used in many studies [3], [17], [18], [19], [20], [44] and remains a staple of BMI research because of its relative simplicity and efficacy.

As research on BMI decoding methods progressed, attention turned to the Kalman filter [21], [22], [23], [24], [45], [46], which explicitly separates the models of how neural activity relates to produce movements and how these movements evolve over time. The Kalman filter, being a probabilistic method, also provides confidence estimates.

Non-linear models of neural tuning provide a better description of neuronal modulations related to motor parameters, but are more computationally demanding to use. The switching Kalman filter, in which several Kalman filters operate in parallel using different parameters, was a non-linear method shown to be superior to the Kalman filter for BMI decoding by Wu et al. [23]. Another non-linear approach, called the particle filter, sequential Monte-Carlo, or condensation, is a recursive Bayesian estimator based on non-parametric representations of probability distributions and stochastic simulation [39]. Several studies have investigated the particle filter for BMI decoding with a variety of non-linear models for neural tuning: Gao et al. [26], [27], Brockwell et al. [25], Shoham et al. [28]. However, due to the heavy computational burden, online closed-loop BMI using the particle filter has not been reported.

Another class of decoding methods work directly from individual neuron spikes instead of instantaneous firing rate estimates. In this approach, spike trains are modeled as discrete events or point processes and decoding can operate at millisecond time scales. The point process analog of the Kalman filter, using a Gaussian representation for uncertainty in state estimates and an inhomogenous Poisson model of spiking, was derived by Eden et al. (2004a, 2004b) and called the *stochastic state point process filter* (SSPPF) [29], [30]. Barbieri et al. estimated the location of a foraging rat using recordings from CA1 hippocampal neurons and the SSPPF [47]. Truccolo et al. (2005, 2008) analyzed and compared the ability of the SSPPF to estimate several behavioral variables in simulations, monkeys, and humans [31], [32]. Wang et al. (2006) showed that preserving a non-parametric posterior distribution for estimated hand movements using a point process particle filter improves decoding accuracy versus the SSPPF in simulation [48]. Brockwell et al. (2007) used a Markov chain Monte-Carlo procedure for fitting point process filter parameters [49]. However, there has been no implementation of an online, closed-loop BMI which uses a point process filter.

To improve decoding of simple reaching movements, tuning to the goal coordinates of reach trajectories has been used to augment tuning to movement. Kemere et al. (2004) included both movement tuning and target position tuning in a maximum-likelihood filter [50]. Srinivasan et al. (2005, 2006) incorporated the estimated target position of a reaching movement in both the Kalman and point process filter frameworks [51], [52]. Later, Srinivasan et al. (2007) combined tuning to target position, point process inputs, and continuous-value inputs to allow neural spikes and other neural measurements such as local field potentials (LFPs), electrocorticography (ECoG), electroencephalography (EEG), and electromyography (EMG) to be used in a single Bayesian filter [38]. Mulliken et al. (2008) included the target location in the state of a Kalman filter for prediction from posterior parietal cortex [53].

Other techniques have been investigated for decoding of continuous hand movements. Isaacs et al. (2000) used principle components analysis and the nearest-neighbor algorithm [54]. Kim et al. (2003) proposed a competitive mixture of linear filters [55], [56]. Sanchez et al. (2002, 2003, and 2004) and Hatsopoulos et al. (2004) proposed various artificial neural-network based approaches [18], [33], [34], [35], [57]. Shpigelman et al. (2003, 2004, and 2005) used support vector regression and a custom-built kernel called the *spikernel* [58], [59], [60]. Fisher and Black (2006) proposed an autoregressive moving average (ARMA) approach [61], and Shpigelman et al. (2008) demonstrated the kernel autoregressive moving average (KARMA) method with the spikernel in closed-loop BMI [62]. In addition to decoding continuous hand movements, a variety of techniques have been employed for decoding discretized action choices, for example, in the studies of Hatsopoulos et al. [18], Musallam et al. [36], and Santhanam et al. [37].

While there is a large variety of algorithms available for decoding desired movement from neural signals, only our approach and the KARMA algorithm of Shpigelman et al. [62] have incorporated non-linear models of neural tuning in closed-loop BMI.

### Quadratic tuning model

In this study, we explored whether a quadratic model of neural tuning can improve BMI decoding accuracy. Our analysis showed that our quadratic model of neural tuning was significantly more predictive of neuron firing rate than a linear model. We then implemented an unscented Kalman filter which used this quadratic model to infer desired hand movements. The increased spike count prediction accuracy (0.02 dB) and off-line reconstruction accuracy of the (1st order) UKF versus the standard Kalman filter (0.39 dB) and 10 tap Wiener filter (0.25 dB) demonstrates the benefits of our quadratic model. By using the unscented transform, we were able to implement a non-linear filter without resorting to computationally expensive particle filtering techniques.

### Movement history

Our decoding method was further enhanced by incorporating a short history of hand kinematics into the hand movement model. We implemented an n-th order UKF which used the hand movement in the n previous time steps to predict hand movements in the next time step. Adding a short history to the state space had the additional benefit of modeling neural tuning across multiple time offsets simultaneously. When using 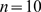 taps, the 10th order UKF produced more accurate reconstruction than the 1st order UKF (0.85 dB improvement), demonstrating the value of incorporating a short history in the state space.

We explored the optimal history length, or number of taps, for the UKF. Our results suggest that the number of taps for best performance depends on the behavioral task. For the pursuit task, accuracy increased with the number of future taps and plateaued at n = 15 or slightly later, and accuracy increased slowly with the number of past taps. For the center-out task, a small number of future taps resulted in the highest accuracy, while the number of past taps had small effects. The improvement of the 10th order UKF versus the 1st order UKF was greater for the pursuit task (mean 1.23 dB) than for the center-out task (mean 0.48 dB).

Based on these results, we conjecture that the richer movement model of the 10th order UKF was able to capture the hand movement patterns produced during the performance of the pursuit task better than those generated during execution of the center-out task. This is likely because hand movements for the center-out task are autocorrelated over shorter time spans than hand movements for the pursuit task. Hand movements during center-out reaches were brief and unrelated between reaches, while during the pursuit task the hand moved relatively smoothly. The n taps of our movement model can be viewed as extra smoothing, hence the pursuit task, with smoother movement trajectories, benefits more than the center-out task. Our analysis showed that the movement model of the 10th order UKF made large contributions to the accuracy of the filter (6.25 vs 3.88 dB in pursuit task), yet this movement model was unable to provide accurate estimates by itself, without the aid of the neural recordings (dead reckoning, Figure 6B).

In previous studies on the Kalman filter, one lingering question was how to set the best time offset in the model between hand movements and neural activity [22]. Wu et al. (2006) searched for the best time offset using a greedy stochastic search mechanism [24]. Our n-th order implementation allowed multiple time offsets to be used simultaneously. The ridge regression regularization used during parameter fitting automatically chooses the best time offset(s) by suppressing the weight coefficients of less useful time offsets. By using regularization, we have essentially replaced the combinatorial search for the best time offset for each neuron with a continuous optimization problem, at the cost of increased bias. We indirectly gauged the benefit of modeling tuning relationships across multiple time offsets by comparing the 1st and 10th order unscented Kalman filters with movement models assumed to be the physical equations relating position and velocity, instead of fitted to training data. The large difference in accuracy (around 2.3 dB) showed the benefit of modeling tuning relationships across multiple time offsets, though much of this improvement is also captured by data-fitted movement models.

### Advantages of the n-th order unscented Kalman filter

The 10-th order and 1st order UKF both produced significantly more accurate reconstructions than the standard Kalman filter, Wiener filter, and the population vector method [10], [13]. In online, closed-loop BMI operation, the 10th order UKF allowed the monkey to perform a pursuit task significantly better than both the Kalman filter (mean improvement 1.04 dB) and Wiener filter (mean improvement 2.49 dB). While the SNR values reported in this study may seem low compared to filter performance in other domains, the large inherent noise in neural activity (compare 0.05 dB mean predictive accuracy per neuron with the accuracy of sensors from other domains) make the BMI decoding problem challenging.

These results demonstrate the advantage of the non-linear model of neural tuning to arm movements at multiple time offsets and the advantage of leveraging patterns of movement. We have demonstrated one computational approach that can achieve these improvements without resorting to a computationally heavy particle filter, the filter design typically used for non-linear observation models. One may argue that the heavy cost of particle filters is not a significant obstacle due to the rapid improvement of computing power, for example, in personal desktop computers. However, an ideal BMI-driven prosthetic device will need to be highly mobile, placing strict limits on power consumption and weight, thus limiting computational power. While modern portable personal computers may be fast enough to host particle filters, they also consume dozens of watts of power and only manage a few hours on a typical battery pack. Thus, an accurate yet computationally efficient filtering algorithm is desirable for a compact BMI-driven prosthetic device.

When compared to the commonly-used Wiener filter, our approach has several advantages. When the parameters of the Wiener filter are fitted using least squares, the noise of the neurons is assumed to be independent and of the same variance. These assumptions are violated by real neural populations [24]. The UKF explicitly models the noise of neurons in a full covariance matrix, allowing different variances among neurons and excess covariance among neurons not due to the desired output variable [24] to be modeled. The Wiener filter typically requires more parameters to be fitted than the UKF, leading to increased training data requirements and increased risk of overfitting. However, overfitting can be mitigated with regularization techniques such as ridge regression or sophisticated Bayesian regression techniques such as Variational Bayesian Least Squares [63]. In contrast to the Wiener filter, the UKF is a Bayesian technique which explicitly models the uncertainty of hand kinematics estimation, giving users access to measures of confidence in kinematic estimates. Furthermore, the UKF explicitly separates the neural tuning model and the movement model. Besides theoretical elegance, this separation allows parameter fitting schemes which can make better use of training data. For example, the model for neural tuning may be estimated from data obtained while the user is performing several different tasks, while individual movement models are estimated for each task. Attempting this with a Wiener filter will confound the autocorrelations from hand movements with the cross-correlation between hand movements and neural activity.

Compared to the point process based methods, our approach offers less temporal resolution. However, the increased temporal resolution offered by point process methods comes at higher computational cost. The normally-distributed noise assumption inherant in all Kalman filters is likely violated by some neurons with such low firing rates that their spike counts per bin are very low. This is one of several approximations made for computational convenience in the Kalman filter approach and a main reason for the development of point process methods. However, point process methods assume all neurons are well discriminated single units, an assumption which is difficult to verify and which forces multiunits to be discarded. To model covariance of the noise among neurons, point process methods must model neuron interactions, which further increase their computational cost or approximation, while neuron noise covariance is included in the basic Kalman filter. For real-time operation on mobile devices, approximations and assumptions of convenience will likely be made by any approach, and the best algorithm will be the one which has the most appropriate tradeoff between accuracy and computational speed.

The switching Kalman filter proposed by Wu et al. [23] is the algorithm most similar to our UKF in design. The switching Kalman filter can be thought of as using a piecewise-linear model, where the pieces are combined in a weighted manner. The space of piecewise-linear functions is clearly more expressive than the space of quadratic functions, but the number of pieces required to approximate quadratic tuning functions for each neuron over many input dimensions (position, velocity, history taps) is very large. Wu et al. reported an approximately 8.9% reduction in mean squared error versus the standard Kalman filter, corresponding to about 0.37 dB improvement. In comparison, our 1st order UKF outperforms the standard Kalman filter by about 0.39 dB and the 10th order UKF outperforms the standard Kalman filter by about 1.25 dB.

The kernel autoregressive moving average (KARMA) algorithm proposed by Shpigelman et al. [62] is the algorithm most similar to our algorithm in capability. Shpigelman et al. used a kernel transform custom-built for neural tuning, called the spikernel, as the kernel for the KARMA algorithm. This kernel allows non-linear, non-parametric tuning models to be used for decoding. The KARMA algorithm, the kernel-trick extension of the well-known ARMA algorithm, also employs an autoregressive movement model to improve predictions. Like our approach, the approach by Shpigelman et al. has achieved real-time, closed-loop BMI operation with a non-linear and pattern-exploiting method. Unlike our approach, the KARMA algorithm is not Bayesian and does not directly produce confidence estimates of its output.

### Future clinical applications

Our n-th order unscented Kalman filter is particularly suited for use in cortically driven prosthetic devices because of its relatively high accuracy and unique features. Our algorithm takes advantage of a non-linear model of neural tuning in a computationally inexpensive implementation that is well suited for mobile, low-power prosthetic systems. Furthermore, our algorithm takes advantage of patterns of movement, abundantly found in typical tasks such as feeding, that a prosthetic may be engaged to do. Since this new approach is Bayesian, it allows the computation of the certitude of decoded movements. Thus, decoded movements with low probaility can be suppressed, and undesired movements caused by decoding errors or unexpected neural activity can be detected and prevented. The separation of the neural tuning and movement models also allows training data to be used more efficiently, making the prosthetic easier to calibrate.

The unscented Kalman filter can be applied to learn neural tuning model parameters or adapt to time-varying neural tuning and time-varying patterns of movement through a technique called dual Kalman filtering for joint parameter and hidden state estimation [64]. Using this approach, a person with paralysis can be trained to use a BMI-driven cortical prosthetic. The user first observes example movements performed by a technician or computer algorithm. Neural activity recorded from the patient's brain and the example movements are then used to compute a first estimate of the neural tuning model. Next, the user assumes the control of the BMI. Then, the UKF would simultaneously decode neural activity and improve the estimates of the neural tuning model parameters. As neural tuning changes over time due to learning, the UKF would modifiy the neural tuning model to exploit these changes. Unlike the co-adaptive framework of Taylor et al. (2002), the UKF would compute in a probabilistically optimal fashion, without requiring knowledge of what the user is doing, and would update models in the background without explicit recalibration, making the system more user friendly.

The UKF can also compensate for degradation of neural recordings as this can be described as changes in the neural tuning model. Furthermore, models of movement can be improved over time to best predict movements produced during execution of particular tasks. These models can also be learned over time to handle novel tasks. Our future work will pursue these approaches toward the development of user-friendly, computationally efficient, and accurate algorithms for BMIs.

## Materials and Methods

### Neuronal recordings

All surgical and experimental procedures conformed to the National Research Council's Guide for the Care and Use of Laboratory Animals (1996) and were approved by the Duke University Animal Care and Use Committee. Cortical recordings were collected from 2 rhesus monkeys (*Macaca mulatta*) performing reaching tasks by moving a computer cursor using a hand-held joystick and by controlling the cursor directly through their cortical activity decoded by a BMI (Figure 1). Monkey C (which performed the task with its left hand) was implanted with four 32-microwire arrays in M1, PMd, PP and supplementary motor area (SMA) in the right hemisphere. Monkey G (which performed the task with its right hand) was implanted with six microelectrode arrays (32 microwires in each) in primary motor cortex (M1), primary somatosensory cortex (S1) and dorsal premotor cortex (PMd) of both hemispheres. Within each array, electrodes were grouped into 16 pairs. The separation between adjacent pairs was 1 mm. Each pair consisted of two microwires placed tightly together with one electrode 300 micron longer than the other. The longer electrode in each pair was equal or larger in diameter. Monkey C was implanted with stainless steel and tungsten electrodes of 46 and 51 micron diameter in areas SMA and M1 and tungsten electrodes of 51 micron diameter in areas PMd and PP. Monkey G was implanted with stainless steel electrodes of 40 and 63 micron diameter (Figure 1B).

The sites with the best quality of neuronal signals were selected. Data from Monkey C were recorded from left PMd (9 daily recording sessions), left SMA (9 sessions), left M1 (9 sessions), and right PP (1 session). Data from Monkey G were recorded from left PMd (13 sessions), left M1 (13 sessions), left S1 (8 sessions), and right PMd (7 sessions). Extracellular neural signals were amplified, digitized, and high-pass filtered using Multichannel Acquisition Processors (Plexon, Inc.). Neuronal action potentials were discriminated by thresholding and sorted on-line through waveform templates set by the experimenter using Plexon spike-sorting software or using templates produced by custom-built spike sorting software [65]. This custom spike sorting software clusters waveforms by their three largest principle components using a modified expectation-maximization algorithm and removes spurious clusters by thresholding on various criteria [65]. Single and multi-units were not treated differently for prediction purposes.

### Behavioral Tasks

During the experimental sessions, each monkey sat in a primate chair. Their heads were unrestrained, and the recording system was connected to the implants using light flexible wires. A two degree of freedom (left-right and forward-backwards) analog joystick was mounted vertically at the monkey's waist level. The joystick was 30 cm in length and had a maximum deflection of 12 cm. The monkeys were trained to manipulate the joystick with their hands. Monkey C performed with the left hand, and Monkey G performed with the right hand. An electrical resistance-based touch sensor on the joystick handle measured whether the monkey was holding the joystick. An LCD projector projected visual images on a screen mounted 1.5 m in front of the monkeys (Figure 1A). Using the joystick, monkeys moved a round cursor, defined by a ring 1.6 cm in diameter. Forward, backward, rightward, and leftward movements of the joystick translated to the upward, downward, rightward, and leftward movements of the cursor, respectively. The joystick to cursor gain varied between 3.2 and 6.4, depending on session (i.e. a 1 cm movement of the joystick translated into a 3.2 to 6.4 cm movement of the cursor). Targets were defined by rings 16 to 20.8 cm in diameter on the screen. The median speeds at which monkeys moved the joystick were approximately 3.5 to 5.5 cm/s, depending on the session.

Each behavioral task required placing the computer cursor over the target using the joystick. The monkeys performed two tasks: (1) center-out and (2) pursuit. The center-out task (Figure 1C) used stationary targets that occurred at randomly chosen points on a fixed-radius ring around the center of the screen. The monkey had to hold the cursor at the center target at the screen center. After the center target disappeared and a peripheral target appeared, the monkey had to move the cursor to the peripheral target and keep inside the target until it received a fruit-juice reward. The inter-trial interval that followed a successful trial was 500 ms. The intertrial interval after an error trial was 700 to 1000 ms. Hold times varied per session from 350 to 1050 ms. The trials in which the monkey failed to put the cursor over the target or failed to fulfill the hold requirement were not rewarded. After a trial was finished, the center target appeared again to start the next trial. In our analysis, data collected during the center-out task were treated as a continuous stream and not segmented by trial or movement onset.

The pursuit task (Figure 1D) used a moving target which followed a Lissajous curve:
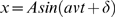
(1a)

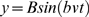
(1b)


where 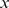 and 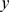 are the x- and y-axis coordinates and 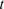 is time in milliseconds. We used parameter values 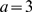, 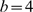, 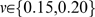 Hz, 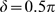, and 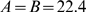
cm (in joystick scale). The temporal frequency was different for the x- and y-axes, making the two coordinates uncorrelated. The monkey had to keep the cursor within the moving target to receive periodic juice rewards.

### Data preprocessing

For all algorithms, spike counts were calculated in 100 ms nonoverlapping bins to estimate the instantaneous firing rate. Joystick position was recorded at 1 KHz and down-sampled to 10 Hz to match the binning rate. Velocity was calculated from position by two-point digital differentiation. Position and velocity data were centered at their means. Spike counts were centered at their means for the Kalman-based filters. Data recorded while the monkey did not hold the joystick were disregarded. Off-line analysis was conducted using MATLAB (Mathworks, Inc). Real-time filters were implemented in a custom built BMI system running on a workstation with an Intel Xeon 2.2Ghz processor.

### Computational Model

Our n-th order unscented Kalman filter (UKF) can be described as a modification of the Kalman filter [42], a commonly-used Bayesian recursive estimation method for a specific class of hidden Markov models (HMMs) with continuous states and observations, normally distributed uncertainty, normally distributed noise, and linear transition and observation models (for more details. An introduction to the Kalman filter can be found in the Supporting Information section (Materials S1). The n-th order unscented Kalman filter combines two extensions: (1) the unscented Kalman filter [41], which allows arbitrary non-linear models to be used in Kalman filtering, (2) the n-th order extension, which allows more expressive autoregressive order n (AR n) movement models and neural tuning models. Figure 2 provides a comparison of the hidden Markov models for the Kalman filter (Figure 2A) and the n-th order unscented Kalman filter (Figure 2B). An example of a linear neural tuning model is shown in Figure 2C, and an example of a quadratic neural tuning model is shown in Figure 2D. Figure 2D depicts example autoregressive (AR 1 vs AR n) movement models (Figure 2D).

In the hidden Markov model for BMI decoding using the n-th order unscented Kalman filter (Figure 2B), the hidden state is the position and velocity of the desired hand movement, described by the variable 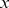. The state transition model or *movement model*, a linear function 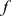, predicts the hidden state at the current time step *t* given the state at the previous *n* time steps:
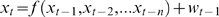
(2)


where 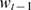 is normal, i.i.d. noise, called the *movement model noise*, which describes the uncertainty arising from approximations made in the model and intrinsic randomness in the movement process. This movement model is an autoregressive process of order n (AR n), as compared to the AR 1 movement models of the Kalman filters previously used for BMI decoding (Figure 2D) [21], [22], [23], [24]. Note that the standard unscented Kalman filter allows non-linear movement models, but we did not design a non-linear movement model and instead focused on a non-linear observation model, described next.

The *observation model* relates the observations to the state via a non-linear function 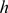:
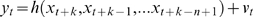
(3)


where 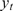 are the observations (100 ms binned spike counts) at time 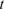 and 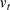 is normal, i.i.d. noise, called the *observation model noise,* which describes the uncertainty in the neural tuning model and the intrinsic randomness of the neurons. The observation model predicts the expected neural activity for a given hand movement state. Following neurophysiological convention, we call it the *neural tuning model*.

We incorporate multiple taps of both position and velocity in the neural tuning model to relate neural activity with hand kinematics at multiple time offsets simultaneously, avoiding the need to search for a best time offset [22], [24]. Note that the neural tuning model captures relationships between neural activity at time 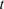 and movements from 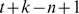 up to time 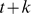, meaning that during decoding, desired movement in the future is predicted. We call the number 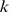 the number of *future taps* and 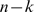 the number of *past taps*. In practice, the predictions into the future are usually inaccurate, but as they pass through the time-tap structure of the filter, they are improved by incorporating information from more neural observations. In all experiments, we used the state tap 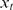 corresponding to the current observations 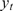 as the filter output, i.e. we did not use lagged estimates or future predictions.

### Quadratic Neural Tuning Model

Many models have been proposed to describe the relationship between neural activity and arm movement, notably the cosine tuning model [43], tuning to speed [31], [66], [67], and tuning to the distance of reach [68], [69]. We used a more general model which we call the *quadratic model of tuning* that combined several features used in the previously proposed models: tuning to position, velocity, distance, and speed. In Cartesian coordinates, the model is:
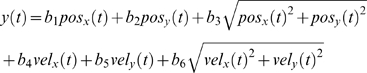
(4)


where 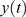 is the mean-subtracted single-neuron firing rate at time 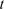, 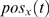 and 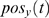 are the x and y coordinates of the cursor at time 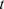, 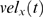 and 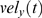 are the x and y velocities of the cursor, and 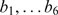 are scalar parameters, one set per neuron. Note that this equation describes the quadratic neural tuning model for the 1^st^ order UKF. For higher values of n, additional terms for the other time offsets are added. For example, the 2^nd^ order UKF with 1 future tap and 1 past tap has a set of terms duplicated with time 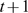. In general, our quadratic model has 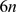 scalar parameters per neuron.

This quadratic model worked well for our experimental task in which the movements were performed by a joystick where the zero position corresponded to the center of the video screen. We chose not to include higher derivative terms, such as acceleration and jerk, because they did not contribute substantially to decoding accuracy.

### Implementation

We implemented the n-th order UKF in Matlab and C++ using the equations presented by Julier et al. [41] with one exception: we used a linear movement model, which meant the first step was the same as that in the standard Kalman filter [42].

The variables in the algorithm are as follows. The vector 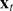 of length 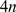 contained the means of the history of state variables at time 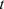:
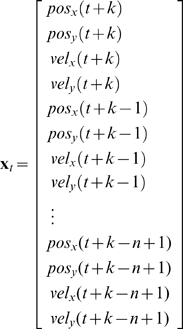
(5)


The 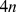 by 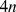 matrix 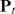
was the state variable covariance matrix. The vector 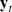 of length *N* was the observed binned spike counts at time 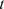, where *N* is the number of neurons.

An iteration of the filter began with the prediction step, in which the state at the previous time step was used to predict the state at the current time step:
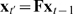
(6)

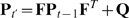
(7)


where 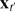
and 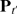 were the mean and covariance of the predicted state, 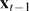
and 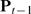 were the mean and covariance of the previous state, matrix 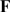 implemented the linear movement model, and 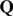 was the covariance of the movement model noise. 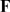 and 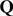 are square 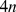 by 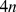 matrices. Details on how these and other parameter matrices were fitted are described in the next section. Besides predicting position and velocity from previous values, the matrix 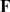 implemented the propagation of taps through time.

Next, the update step corrected the prediction from the prediction step using the observations in a Bayesian way. In the Kalman filter, the neural tuning model is linear and the update step can be implemented in a series of matrix equations (Table 1) [42], because linear models allow straightforward, closed-form computation of the posterior distribution of the state estimate given the observation. However, analytical calculation of the posterior distribution is, in general, only possible under this linear model assumption [70]. For arbitrary non-linear observation models, computing the posterior distribution poses an intractable integration problem [70]. The unscented Kalman filter gives an approximate solution using the unscented transform — a method for approximating the mean and covariance of normally distributed random variables after they have passed through a non-linear function [41]. This transform uses a fixed set of algorithmically selected simulation points, called *sigma points*. The sigma points completely capture the first and second moments of the distribution [70]. Geometrically speaking, the sigma points are located at the mean and along the eigenvectors of the covariance matrix, if the orthgonal matrix square root is used in their calculation [41], though we used the Cholesky decomposition for the matrix square root. 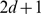 sigma points are required, where 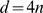 is the dimension of the state space. The set of sigma points is calculated from the state mean and covariance and evaluated through the non-linear observation function. The mean and covariance of the result are then calculated by taking the weighted mean and weighted covariance of the sigma points (for a detailed review see [70]). This approximation scheme computes precisely the effect on the mean and covariance of a normal distribution by the third order and below terms of the Taylor expansion of the non-linear function, while presence of fourth order or higher terms in the Taylor expansion introduce error [70]. Since we use a quadratic observation function, the mean and covariance of our predicted observations are calculated precisely by the unscented transform. However, the non-linear observation function makes the distribution of the predicted observation no longer normal, while the unscented Kalman filtering paradigm assumes normality and discards the higher order moments, introducing approximation error. Compared to the extended Kalman filter (EKF) [42], a well-known non-linear filtering technique, the unscented Kalman filter has better approximation accuracy for the same asymptotic computational cost [70].

In the general unscented Kalman filter, the sigma points are generated from 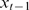 and 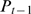 and evaluated in the non-linear state transition and observation functions. In our implementation, only the neural tuning model was non-linear, so sigma points were generated from 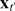 and 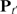. The sigma points 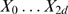 were set as:
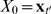
(8a)

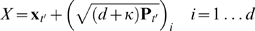
(8b)

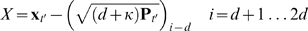
(8c)


where the subscript outside the parentheses indicate the row taken from the matrix inside the parentheses. The square root is the matrix square root. For robustness, this computation was performed using the Cholesky decomposition. 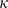 is a parameter which specifies how heavily the center sigma point is weighted compared to the other sigma points. Adjusting this parameter can improve the approximation of higher order moments [70]. We used the conventional value of 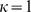 for normal distributions.

Next, the sigma points were evaluated in the quadratic neural tuning function 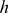:
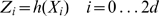
(9)


where 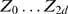
denote the sigma points after observation function evaluation. These function evaluations were implemented as 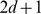
separate matrix multiplications of the form:
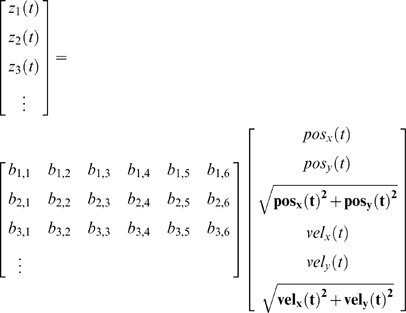
(10)


where 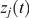 is the predicted (mean-subtracted) spike count for neuron 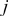 at time *t*, the vector on the left hand side is one post-function sigma point 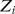, and the right-most vector is one pre-function sigma point 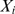 with augmented terms. The bolded augmented terms are added to each of the sigma points using the sigma points' own values for position and velocity. Note equation 10 shows the multiplication for the 1^st^ order UKF. For higher n, there are more columns of model parameters in the parameter matrix and more rows in the vector 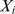 corresponding to the history taps. The 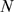 by 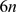 matrix in the center of equation 10 containing the neural tuning model parameters, 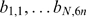, for all *N* neurons is called matrix 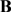, which has a similar function to matrix 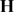 of for the Kalman filter.

The mean and covariance of the predicted neural firing rates were found using weighted mean and weighted covariance:
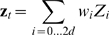
(11)

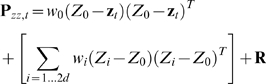
(12)


where 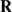
is the covariance matrix of the tuning model noise. The weights were:
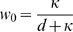
(13a)

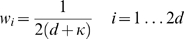
(13b)


Then, the Kalman gain was calculated: 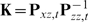
(14)


where the state-observation cross-covariance 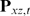, was:
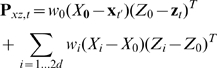
(15)


The Kalman gain was used to correct the state estimate using the discrepancy between the predicted and actual (mean-subtracted) spike counts:
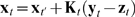
(16)


Finally, the state covariance was updated: 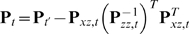
(17)


Equations 6 through 17 implement one iteration of the algorithm. A side-by-side comparison of the equations for the Kalman filter and the n-th order unscented Kalman filter are shown in Table 1.

In off-line reconstructions, the initial values of 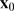 were set by taking the means of the state variables in the training data, and the initial values of 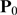 were set by taking the covariance of the state variables in the training data. When n was larger than 1, the means and covariances for the initial values were duplicated for each tap, so that the initial covariance matrix had a block-diagonal form with n blocks. In on-line BMI, the initial values of 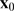 were set as the joystick position and velocity at that time and initially the values of 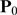 were set to the identity matrix corresponding to variance of 1 cm for position and 10 cm/sec for velocity.

### Parameter Fitting

We fitted the parameter matrices 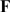 and 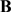 to training data using regularized linear regression and estimated the matrices 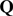 and 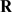 from the regression residuals. We chose a form of Tikhonov regularization called ridge regression because of its simplicity and low computational cost.

To fit 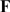, we first composed the 4 by *T* matrix 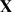 of the training data position and velocities, where *T* is the number of data points (i.e. the time length of the training data). We then constructed a 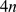 by *T* matrix 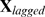, where column *i* of 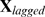 was the vertical concatenation of columns 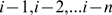 of matrix 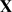. To avoid the missing data problem when filling the first *n* columns of 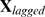, the first *n* columns of 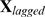 and 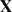 were omitted when fitting 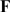. Then, we fitted the intermediary matrix 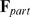 using ridge regression:
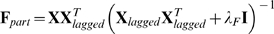
(18)


where 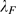 was the ridge regression parameter. The selection of ridge regression parameters is discussed in the next section. 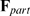 was then augmented with entries which propagated the history taps to make 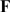:
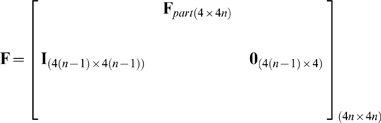
(19)


where 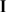 is the identity matrix and 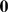 is the zero matrix. Subscripts indicate matrix sizes.

An alternative method for setting the movement model is to use the equations describing motion, e.g. position is the integral of veloctiy over time. However, this method does not capture the patterns in the movements generated by the BMI user as well as movement models fit from kinematic data. In practice, our fits to 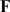 are similar to the matrix implementing the motion equations except for modest perturbations.

The movement model noise covariance matrix 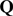 was estimated by first computing 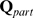:
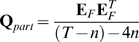
(20)


where 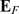 is the 4 by 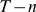 residual matrix from fitting 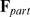, and the division is executed per element. We then augmented 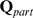 to construct 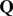:
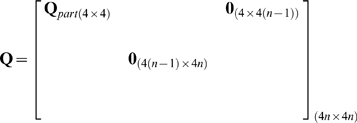
(21)


To fit 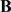, we first constructed the *N* by *T* matrix 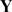 of mean-subtracted binned spike counts from the training data, with the spike counts from all neurons at one time step in each column. We then constructed the 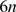 by *T* matrix 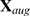, where column *i* of 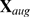 was the vertical concatenation of columns 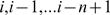 of matrix 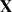 with the bolded quadratic terms in equation 10 inserted appropriately. To implement the *k* future taps, 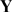 must be shifted back in time by *k* steps. This was done by removing the last *k* columns of 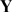 and the first *k* columns of 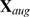. Subsequently, to avoid the missing data problem when filling the first 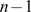 columns of 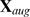, the first 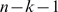 columns of 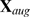 and 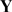 were removed. We then fitted 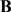 using ridge regression:
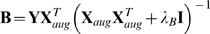
(22)


where 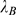 was the ridge regression parameter.

The *N* by *N* neural tuning model noise covariance matrix 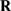 was estimated using:
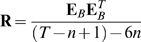
(23)


where 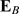 is the *N* by 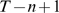 residual matrix from fitting 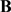, and the division is executed per element.

### Algorithm Evaluation

The n-th order unscented Kalman filter and several comparison methods were evaluated off-line using data collected in experiments in which monkeys moved a computer cursor using the joystick. The n-th order UKF used 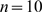 taps, with five future taps and fivepast taps. The UKF with 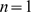 past taps was tested to evaluate the benefit of 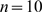 taps. A standard Kalman filter was evaluated to determine the benefit of the quadratic tuning model. For comparison against algorithms commonly used for a closed-loop BMI, a Wiener filter with 10 taps and the population vector method used by Taylor et al. [13] were evaluated.

For off-line reconstructions, cross-validation was conducted. In this procedure, a portion of the data for each session was held-out for testing and the rest was used to fit parameters. Performance of the algorithms was evaluated on the held-out portion to avoid fitting models and making predictions on the same data. The data for each session were divided into 10 equal-sized portions (or *folds*) and the testing procedure was repeated on each held-out portion in turn. Both the movement and neural tuning models were fit for each cross-validation fold. In this study we did not address the question of how to design a general movement model, instead we leave this for future work.

For off-line reconstructions, ridge regression parameters for every algorithm fitted using ridge regression were chosen by optimizing for highest position reconstruction accuracy on the first cross validation fold of each session, i.e. fitting and predicting was performed repeatedly for different choices of 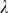 (for the UKF, 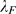 and 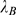 were sought independently) on the first cross validation fold. This first fold was omitted when aggregating performance metrics. For on-line experiments, ridge regression parameters were set to 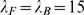
for the 10^th^ order UKF, 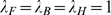 for the 1^st^ order UKF and Kalman filter, and 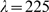 for the Wiener filter. These values were picked using previous experience. Wiener filter parameters were also fit with ordinary least squares (OLS) without ridge regression to demonstrate the benefit of regularization.

The Kalman filter used for comparison had the same state variables as the 1^st^ order UKF, and its models were fitted in a similar way as the 1^st^ order UKF, less the quadratic terms for the observation model matrix (see Supplementary Materials).

For the population-vector method, the neuronal weights were fit via ordinary least squares without regularization. The original formulation of the population vector method predicted velocity and did not predict position directly. To make position predictions, we substituted the Cartesian position coordinates for the velocity components. We implemented the Taylor et al. [13] version of the population vector method with one slight modification: the baseline firing rate (mean) and normalization constant (standard deviation) of neurons were fit once from training data, instead of updated during filtering using a sliding window of spiking history.

To quantify filter performance, we compared algorithm estimated trajectories to joystick trajectories (in off-line reconstructions) and to target trajectories (in closed-loop BMI). We computed two metrics: the signal-to-noise ratio (SNR) and the correlation coefficient (CC). SNR was calculated as:
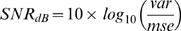
(24)


where 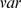 is the sample variance of the desired values (joystick or target) and 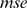 is the mean squared error of the predicted values from the desired values. Position, velocity, and the x and y axes were evaluated separately. The signal-to-noise ratio can be viewed as the inverse of the normalized mean squared error, where the normalization factor quantifies the power of the desired signal. SNR is widely used in engineering and has been previously used to measure BMI decoding performance [34], [44]. The SNR is unitless and comparable across experimental setups, unlike the mean squared error, which is usually incomparable between studies due to differences in movement magnitudes. In this respect the SNR is similar to the CC. However, the SNR is *not* translation and scale invariant, unlike the CC. This is an advantage because translation and scale invariance imply that the CC may leave undetected certain unwanted filtering results. For example, a predicted hand trajectory that is incorrect by a large but constant displacement has the same CC as a trajectory without the erroneous displacement, since only deviations from the mean are analyzed by the CC. As indicated by its name, CC is a measure of correlation, but we are interested in measuring accuracy. Furthermore, as the CC saturates at 1, its scale is compressed as it approaches 1, making it more difficult to grasp intuitively and making similar increments at lower values of CC and higher values of CC incomparable. Short of benchmark datasets, we believe the SNR measure best facilitates direct comparison between algorithms developed by different authors.

To aggregate results for each session, mean SNR and CC among the cross-validation folds and between the x- and y-axis predictions were computed. Standard error of the mean was calculated for each session with 18 observations (9 folds×2 axes). To test for significant effects, we treated each cross-validation fold and each axis as a condition for paired, two-sided sign tests. We used an 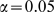 significance level.

## Supporting Information

Materials S1 (0.26 MB DOC)
