# Supplementary material for: Unscented Kalman Filter for Brain-Machine Interfaces
Source: PLoS One. 2009 Jul 15;4(7):e6243. doi: 10.1371/journal.pone.0006243 (PMC2705792; doi:10.1371/journal.pone.0006243)
Supplement: Materials S1 — (0.26 MB DOC) [file pone.0006243.s001.doc]

**SUPPLEMENTARY MATERIALS**

**Unscented Kalman Filter for Brain-Machine Interfaces**

Zheng Li^1^, Joseph E. O'Doherty^2^, Timothy L. Hanson^3^, Mikhail A. Lebedev^3^, Craig S. Henriquez^2,5^, Miguel A. L. Nicolelis^2,3,4,5,6,*^

Depts. of Computer Science^1^, Biomedical Engineering^2^, Neurobiology^3^, Psychology and Neuroscience^4^, Duke Center for Neuroengineering^5^, Duke University, Durham, NC;

Edmond and Lily Safra International Institute of Neuroscience of Natal^6^, Natal, Brazil.

Running head: Unscented Kalman filter for brain machine interfaces

*Corresponding Author:

Room 327 Bryan Research Building

Box 3209 Department of Neurobiology

Duke University

Durham, NC 27710

(919) 684-4580

nicoleli@neuro.duke.edu

*Kalman Filter*

Enumeration of equations continues from the Materials and Methods section of the main manuscript. In the Kalman filter hidden Markov model, the variables in the system are separated into hidden variables, called states, and observable variables, called observations. Time is discretized into equal fixed-duration steps. For BMI, the hidden state is a behavioral parameter being decoded (e.g., the hand or cursor position) and the observations are measurements of neural activity. Statistical uncertainty in the variables is represented by a multi-variate normal distribution in which the vector of mean values and the covariance matrix fully describe the variables' joint probability distribution. In our behavioral task, the state variables at time $t$ are represented by a column vector :

$\mathbf{x}_{t}=\left[\begin{matrix} pos_{x}(t) \\ pos_{y}(t) \\ vel_{x}(t) \\ vel_{y}(t) \end{matrix}\right]$ (25)

where $pos_{x}(t)$ and $pos_{y}(t)$ are the x- and y-axis position coordinates of the cursor at time *t* and $vel_{x}(t)$ and $vel_{y}(t)$ are the x- and y-axis velocities at time *t*. Statistical uncertainty in the state is described by the state covariance matrix $\mathbf{P}_{t}$. Neuronal firing rates at time $t$ are represented by a column vector $\mathbf{y}_{t}$. The instantaneous firing rates are estimated by binning spike counts (bin size = 100 ms, non-overlapping) of a population of neurons.

A linear function $f$, called the *movement model*, predicts the mean of the state at the current time step given the mean of the state at the previous time step:

$\mathbf{x}_{t}=f(\mathbf{x}_{t-1})+\mathbf{w}_{t-1}$ (26)

where $\mathbf{x}_{t}$and$\mathbf{x}_{t-1}$ are the states at time steps *t* and *t-1*, respectively, and $w_{t-1}$ is normally distributed noise, called the *movement model noise*, that describes the uncertainty arising from approximations made in the model and intrinsic randomness in the process. The function $f$ describes how the state (hand or cursor position and velocity) is expected to change from one time step to the next, before any information from observing the neural activity is taken into account. Thus, the movement model *predicts* the state at the next time step using the current state. This prediction is then corrected or *updated* using observations of the neural activity and the *observation model*.

In the *observation model,* the observations (i.e., measurements of neural activity), $\mathbf{y}_{t}$, are related to the state, $\mathbf{x}_{t}$, via a linear function $h$:

$\mathbf{y}_{t}=h(\mathbf{x}_{t})+\mathbf{v}_{t}$ (27)

where $\mathbf{y}_{t}$ is the column vector containing (mean subtracted) neuronal rates at time *t* and $\mathbf{v}_{t}$ is normally distributed noise, called the *observation model noise,* which describes the uncertainty in the neural tuning model. The observation model predicts the expected neural activity for a given state, and we call it the *neural tuning model*.

The Kalman filter iteration at each time step consists of two steps, the prediction step and the update step, which correspond to the movement (equation 26) and observation models (equation 27) (for details, see Haykin, 1996). These steps are implemented as matrix equations.

The *prediction step* generates a prediction of the position and velocity of the hand at the next time instant using the movement model. This is "dead reckoning" based solely on prior movements, without the aid of neural recordings:

$\mathbf{x}_{t'}=\mathbf{Fx}_{t-1}$ (28)

where $\mathbf{x}_{t'}$ is the predicted state, $\mathbf{x}_{t-1}$is the preceding state, and matrix $\mathbf{F}$ implements the linear movement model (compare to equation 26). $\mathbf{F}$ is a square $d$ by $d$ matrix, where $d$ is the number of state variables. In our Kalman filter implementation, $d=4$ for the x- and y-axis positions and velocities.

Since $\mathbf{x}_{t'}$ is a guess of the current state which may be inaccurate, the uncertainty in the estimate needs to be modified. Accordingly, the prediction step includes a computation that modifies the state covariance matrix:

$\mathbf{P}_{t'}=\mathbf{FP}_{t-1}\mathbf{F}^{T}+\mathbf{Q}$ (29)

where $\mathbf{P}_{t-1}$is the state covariance at the previous time step, $\mathbf{P}_{t'}$is the state covariance after the prediction step, and $\mathbf{Q}$ is the $d$ by $d$ covariance matrix of the movement model noise. Pre- and post-multiplying the state covariance $\mathbf{P}_{t-1}$ by the matrix $\mathbf{F}$ modifies the uncertainty in our state estimate according to the movement model in a probabilistically-correct way. Adding the matrix $\mathbf{Q}$, representing the zero mean, normally-distributed movement model noise $\mathbf{w}_{t}$, increases the state covariance to account for the uncertainty introduced by the "dead reckoning".

In the *update step*, the model of neural tuning is applied to the predicted hand position and velocity to generate predicted firing rates for each neuron:

$\mathbf{z}_{t}=\mathbf{Hx}_{t'}$ (30)

where $\mathbf{z}_{t}$ is the predicted binned firing rates of the neurons, $\mathbf{x}_{t'}$ is obtained from equation 28, and $\mathbf{H}$ is the matrix that implements the linear neural tuning model (compare to equation 27). $\mathbf{H}$ is a *N* by *d* matrix, where *N* is the number of neurons.

The difference between the predicted neuronal rates $\mathbf{z}_{t}$ and the actual rates $\mathbf{y}_{t}$ is used to correct the predicted state $\mathbf{x}_{t'}$ to better correspond to the observed neuronal rates. This correction first requires the calculation of the matrix $\mathbf{S}_{t}$, an $m$ by $m$ covariance matrix that describes statistical uncertainty in the predicted firing rates:

$\mathbf{S}_{t}=\mathbf{HP}_{t'}\mathbf{H}^{T}+\mathbf{R}$ (31)

where matrix $\mathbf{R}$ is the $m$ by $m$ covariance matrix for the zero mean, normally-distributed neural tuning model noise $v_{t}$.

The amount of correction depends on the uncertainty of the current state estimate and the uncertainty in the predicted neuronal rates. For example, if the state estimates have high certitude (small $\mathbf{P}_{t'}$ ) and the predicted neuronal rates are noisy (large $\mathbf{S}_{t}$), the correction should be small and vice versa for large $\mathbf{P}_{t'}$ and small $\mathbf{S}_{t}$. The probabilistically optimal amount of correction given the linearity and Gaussianity assumptions is called the Kalman gain $\mathbf{K}_{t}$:

$\mathbf{K}_{t}=\mathbf{P}_{t'}\mathbf{H}^{T}\mathbf{S}_{t}^{-1}$ (32)

The Kalman gain is used to carry out the correction: $\mathbf{x}_{t}=\mathbf{x}_{t'}+\mathbf{K}_{t}(\mathbf{y}_{t}-\mathbf{z}_{t})$ (33)

where $\mathbf{K}_{t}$ acts as a gain (and the Kalman filter thus called a “filter”) because it allows only a portion of the correction from the observations to update the state estimate. Compared to applying the entire correction, the Kalman gain reduces the adverse effect of neuronal noise on the state estimates.

Finally, the state covariance matrix $\mathbf{P}_{t}$ is updated using the Kalman gain:

$\mathbf{P}_{t}=(\mathbf{I}-\mathbf{K}_{t}\mathbf{H})\mathbf{P}_{t'}$ (34)

where matrix $\mathbf{I}$ is the $d$ by $d$ identity matrix. The operation in equation 34 decreases the state covariance because the observations increase the certitude of the state estimate.

The initial values for the state $\mathbf{x}_{0}$ and state covariance $\mathbf{P}_{0}$ can be set to the true values, if they are available, or otherwise the expectation and covariance of the state in the data used for parameter fitting. We used the expectation and covariance of the training data to initialize for off-line reconstructions, and we used the true values for the joystick position and velocity and the identity matrix to initialize during on-line BMI experiments.

We fitted the matrices $\mathbf{F}$, $\mathbf{H}$, $\mathbf{Q}$, and $\mathbf{R}$ using a segment of the data recorded when the monkeys controlled the cursor with the joystick (*training data*). The training data included both the recorded neuronal rates and the cursor trajectory. (A separate piece of data, called the *testing data*, was used for evaluating the algorithm’s predictions.) The $\mathbf{F}$ and $\mathbf{H}$ matrices were calculated using multilinear regression and the $\mathbf{Q}$ and $\mathbf{R}$ matrices were estimated from the regression residuals.

To calculate $\mathbf{F}$, we composed the recorded states ($pos_{x},pos_{y},vel_{x},vel_{y}$) into a *d* by *T* matrix $\mathbf{X}$, where *T* is the total number of data points (time length of the training data). Each row in $\mathbf{X}$ corresponded to a state variable and each column corresponded to a time step. Then, we constructed a second matrix $\mathbf{X}_{shift}$, where the column $i$ of $\mathbf{X}_{shift}$ was the $i+1$ column of $\mathbf{X}$. $\mathbf{X}_{shift}$ represented the state variables one time step later compared to $\mathbf{X}$. To avoid the missing data problem, we omitted the last columns of $\mathbf{X}$ and $\mathbf{X}_{shift}$ when fitting $\mathbf{F}$. $\mathbf{F}$ is then the least square solution to

$\mathbf{FX}=\mathbf{X}_{shift}$ (35)

To improve the generalization ability of $\mathbf{F}$ and to avoid over-fitting, we solved equation 35 using the Tikhonov regularization technique called ridge regression:

$\mathbf{F}=\mathbf{X}_{shift}\mathbf{X}^{T}(\mathbf{XX}^{T}+\lambda_{F}\mathbf{I})^{-1}$ (36)

where $\mathbf{I}$ is the *d* by *d* identity matrix and $\lambda_{F}$ is the ridge regression parameter.

The movement model noise covariance matrix $\mathbf{Q}$ was estimated using:

$\mathbf{Q}=\frac{\mathbf{E}_{F}\mathbf{E}_{F}^{T}}{T-d}$ (37)

where $\mathbf{E}_{F}$ is the *d* by  residual matrix from fitting $\mathbf{F}$, and the division is executed per element.

To fit $\mathbf{H}$, we constructed a *N* by *T* matrix $\mathbf{Y}$ of the binned neural spike counts with rows corresponding to neurons and columns corresponding to time steps. Columns of $\mathbf{Y}$ were synchronized with columns of $\mathbf{X}$. $\mathbf{H}$ is then the least squares solution to

$\mathbf{HX}=\mathbf{Y}$ (38)

As in the calculation of $\mathbf{F}$ (equation 36), ridge regression was used to improve generalization ability and to avoid over-fitting:

(39)

where $\lambda_{H}$ is the ridge regression parameter.

The neural tuning model noise covariance matrix $\mathbf{R}$ was estimated using:

$\mathbf{R}=\frac{\mathbf{E}_{H}\mathbf{E}_{H}^{T}}{T-d}$ (40)

where $\mathbf{E}_{H}$ is the *N* by *T* residual matrix from fitting $\mathbf{H}$, and the division is executed per element.
